# Robotic Ultrasonic Measurement of Residual Stress in Complex Curved Surface Components

**DOI:** 10.1155/2019/2797896

**Published:** 2019-03-03

**Authors:** Qinxue Pan, Chang Shao, Dingguo Xiao, Ruipeng Pan, Xiaohao Liu, Wei Song

**Affiliations:** ^1^School of Mechanical Engineering, Beijing Institute of Technology, Beijing 100081, China; ^2^Key Laboratory of Fundamental Science for Advanced Machining, Beijing Institute of Technology, Beijing 100081, China

## Abstract

The automatic measurement, especially for products with complex shapes, has always been one of the most important application areas of robots. Aiming at the challenge of measuring residual stress under curved surface, in this paper, the residual stress ultrasonic measuring robot system with two manipulators is constructed, which is based on combining industrial robot technology with residual stress ultrasonic nondestructive measuring technology. The system is mainly composed of a motion control system, an ultrasonic detection system, and a data processing system. The robotic arm controls the movement of the two ultrasonic transducers along the set scanning path which is based on the geometric model of components and adjusts the transducer's posture in time according to the shape of the workpiece being measured. The configuration information based on workpiece coordinate system is transformed into a position data that takes into consideration the first critical angle and can be recognized by the robot. Considering the effect of curvature, the principle model of residual stress measuring by the critical refraction longitudinal wave is established. The measured signal including the stress state of the measured region, as well as the actual position and posture information of the transducers, is processed by the computer in real time, which realizes automatic nondestructive measurement of residual stress under curved surface.

## 1. Introduction

Currently, complex surface components have been widely used in aviation, aerospace, marine, automobile, and molds industry and have played an extremely important role, such as in engine blades and propellers, which is directly related to the reliability and safety of the equipment. The manufacturing process of complex surface components is cumbersome and complex, which will inevitably produce residual stress on the surface [1]. Even after a certain process, for example, heat treatment, the residual stress is also difficult to eliminate completely. On the other hand, these complex surface components will also have residual stresses due to impact loads, thermal loads, and corrosion during long-term service. The existence of this residual stress not only seriously affects the shape and mechanical properties of the components but also promotes the generation and propagation of cracks, causing the components to suddenly break under the working load, leading to malfunctions and even serious safety accidents [2]. Therefore, it is very important to measure and evaluate the residual stress of complex surface components.

Ultrasonic detection technology based on acoustic elasticity principle is one of the reliable and effective methods for residual stress nondestructive measuring. Residual stress ultrasonic measuring technology is more and more widely used in the inspection of residual stress of regular-shaped members such as rails, pipes, gears, and hubs due to its advantages of nondestructivity, harmlessness, reliability, accuracy, and convenience. Duquennoy et al., of the University of Valensina, France, used Rayleigh waves that excited by laser and received by PZT to measure the residual stress on the pipe surface [3]. Using the similar method, Wanwan et al. measured the residual stress in a cast iron brake disc and compared with that measured by X-ray stress analyser [4]. In general, the research hotspots of ultrasonic nondestructive testing of residual stress mainly focus on different application objects and different ways of ultrasonic signal excitation and reception in recent years [5–8].

The residual stress ultrasonic measuring has a strict requirement on the incident and receiving angles of the transducer. However, for a complex curved surface component, the change of curvature will seriously affect the incidence, propagation, and reception of the ultrasonic signal, which poses great challenges to ultrasonic measuring of residual stress in complex surface components. The traditional manual measurement not only is difficult to ensure the necessary position and posture of the ultrasonic transducer but also has disadvantages such as low efficiency, large labor intensity, poor detection accuracy, and difficulty in quantitative analysis.

The development of high-precision, multi-degrees-of-freedom robots has brought a new support to the ultrasonic measurement for complex surface components [9, 10].

In this paper, an ultrasonic residual stress measurement method based on robot technology is proposed, which takes full advantage of a robot's precise control of ultrasonic transducer position and automatic scanning. It adjusts the posture of the ultrasonic transducer in real time according to the detection position. The actual posture information of the ultrasonic transducers and the ultrasonic signal at this position is processed by the computer to obtain the residual stress value of the measured area.

## 2. Materials and Methods

### 2.1. Ultrasonic Measuring Principle of Residual Stress in Curved Surface Components

The main basis for the ultrasonic measurement of stress is the acoustic elasticity theory, that is, the stress state in the elastic solid will affect the propagation speed of the ultrasonic wave in a material [11, 12]. Theoretical and experimental studies show that the ultrasonic longitudinal wave with the propagation direction being consistent with the stress direction is most sensitive to the change of stress. Therefore, it is necessary to generate a longitudinal wave propagating along the surface. By measuring the change of the longitudinal wave speed, we can realize the nondestructive detection of the tangential residual stress state in the propagation direction in the surface layer.

**Figure 1:**
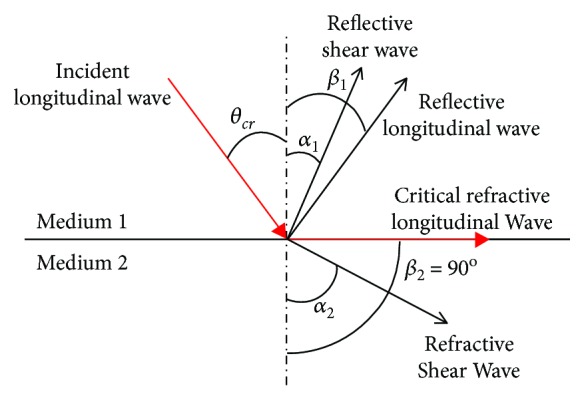
Wave conversion.

As shown in Figure 1, when the longitudinal wave propagates from a medium with a slower wave velocity to a medium with a faster wave velocity (such as from water to metal material), according to Snell's theorem, there is an incident angle so that the refracted longitudinal wave has a refraction angle of 90 degrees. This refracted longitudinal wave will propagate along the surface of the second medium. This incident angle is called the first critical angle (*θ*_cr_), and the resulting refracted longitudinal waves are called critical refraction longitudinal wave (*L*_cr_).

The first critical angle can be obtained by Snell's theorem:

(1) $$\begin{array}{c} \theta_{\text{cr}}=\sin^{-1}\frac{V_{1L}}{V_{2L}}, \end{array}$$

where *V*_1*L*_ is the longitudinal wave velocity of medium 1 and *V*_2*L*_ is the longitudinal wave velocity of medium 2.

**Figure 2:**
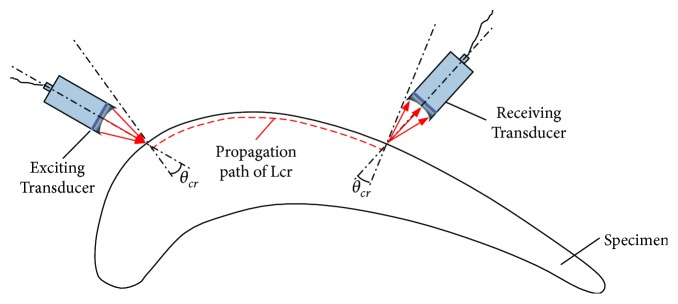
Critical refracted longitudinal waves propagating in a curved surface.

For complex surface components, in order to excite and receive the critical refracted longitudinal wave at the surface of the component, it must be ensured that the ultrasonic transducer is tilted by a certain angle. The angle between the excitation transducer and the normal of incident point is equal to a positive critical angle, and the receive transducer and the normal of exit point is equal to a negative critical angle. It must also ensure that the exciting and receiving transducers are in the same plane as the refracted longitudinal wave propagation path. The excitation, propagation, and reception process of critical refraction longitudinal wave along the surface is shown in Figure 2.

**Figure 3:**
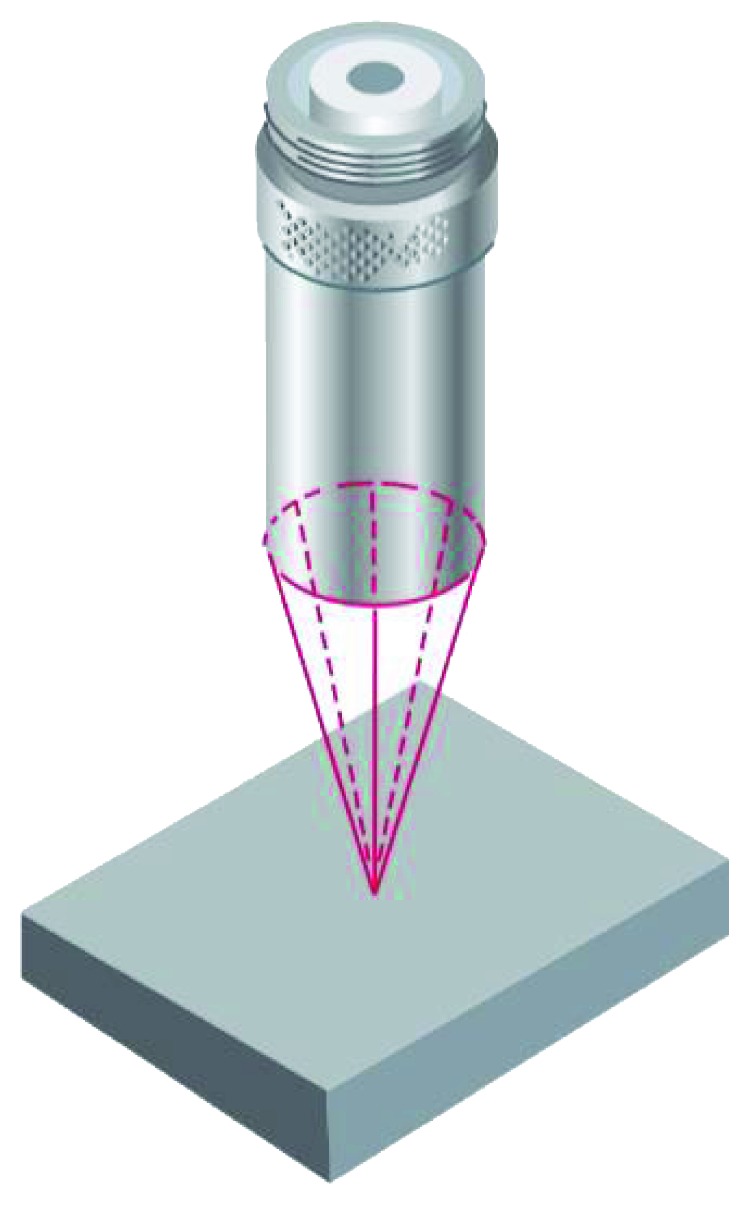
Spherical focus transducer.

In order to overcome the diffusion of the ultrasonic beam in the coupling agent and improve the detection sensitivity and resolution of the curved surface workpiece, the focal transducer is used for detection, which is shown in Figure 3. The focus position of the transducer in water can obtained by calculation or experiment.

According to the principle of ultrasound, the focal length of the transducer in water is calculated as follows:

(2) $$\begin{array}{c} F=\frac{R}{1-C_{2}/C_{1}}, \end{array}$$

where *F*, *R*, *C*_1_, and *C*_2_ are the focal length of the transducer in water, curvature radius of the acoustic lens, ultrasound speed in the lens, and ultrasound speed in water, respectively.

Based on the theory of acoustic elasticity, the relationship between ultrasonic longitudinal wave velocity and stress can be simplified as follows:

(3) $$\begin{array}{c} V_{\text{L}\sigma }=V_{\text{L}0}\left(1-K_{\text{L}}\sigma \right), \end{array}$$

where *V*_L*σ*_ represents the longitudinal wave velocity when the stress is *σ*, *V*_L0_ is the longitudinal wave velocity in the absence of stress, and *K*_L_ is the acoustic elastic coefficient of longitudinal wave.

Suppose that the propagation path is *s*, the propagation time is *t* and the tangential stress on the path is *σ*. Because the velocity is difficult to measure directly, the different time that ultrasound travels the same distance is used to calculate the stress state on the path:

(4) $$\begin{array}{c} \Delta t=\frac{s}{V_{\text{L}0}\left[1-K_{\text{L}}\sigma \right]}-\frac{s}{V_{\text{L}0}}=\frac{{sK}_{\text{L}}\sigma }{V_{\text{L}0}\left[1-K_{\text{L}}\sigma \right]}, \end{array}$$

(5) $$\begin{array}{c} \sigma =\frac{\Delta t\cdot V_{\text{L}0}}{K_{\text{L}}\left(s+\Delta t\cdot V_{\text{L}0}\right)}. \end{array}$$

### 2.2. Trajectory Planning

According to the principle of ultrasonic measurement of residual stress for curved surface components, it must be ensured that the exciting and receiving transducers move along the set path with a specific posture. Therefore, it is necessary to perform trajectory planning on the measured surface to obtain the controlled movement point of the manipulator. Based on the CAD model of the component, the computer-aided manufacturing (CAM) numerical simulation software is used to obtain the position and normal vector of the scanning trajectory points in the Cartesian coordinate system.

**Figure 4:**
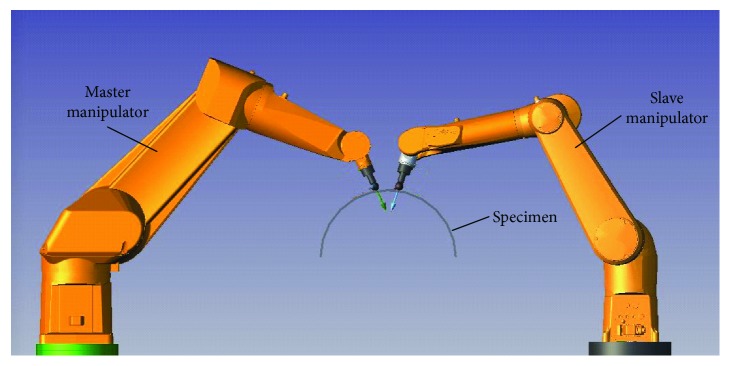
Manipulator distribution.

As shown in Figure 4, the robot arm that holds the exciting transducer is defined as the master manipulator and the robot arm that holds the receiving transducer as the slave manipulator.

**Figure 5:**
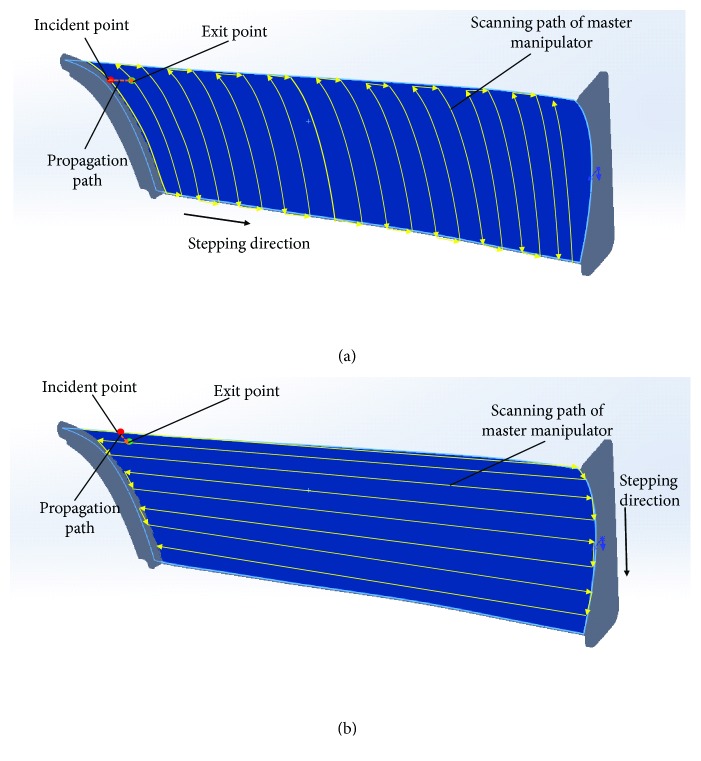
Different zig-zag scan modes for surface workpiece.

Both the master manipulator and slave manipulator move in the zig-zag scanning mode, and the slave manipulator always keeps a certain distance from the master manipulator in the stepping direction, which determines the spatial resolution of the detection.  Figure 5 shows two different zig-zag scan modes for surface workpiece. Through this two different scanning methods, we can get the stress components in two directions, and then according to the principle of force synthesis, we can determine the stress vector in the surface direction.

### 2.3. Coordinate Transformation

Considering the requirements for transducer position orientation, the pose information in the Cartesian coordinate system of workpiece cannot be directly recognized and used by the robot controller. We need to convert that into point and the orientation data of the transducer based on the coordinate system of the robot.

**Figure 6:**
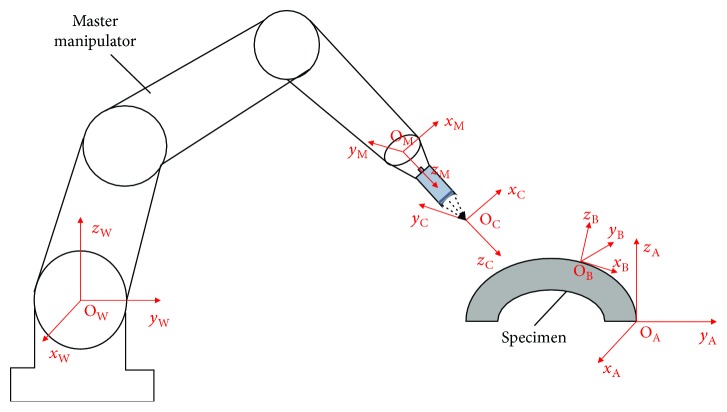
Definition of the coordinate systems in the master manipulator.

As shown in Figure 6, taking the master manipulator motion control as an example, set the reference coordinate system of the robot as {W} and the tool coordinate system as {M}. The tool coordinate system translated along the *z*-axis to the focus of the ultrasonic transducer is {C}, the workpiece coordinate system is {A}, and the measured discrete coordinate system is {B}. The origin of the {B} coordinate system is specified at the scanning point. The *Z*-axis is along the normal direction of the scanning point, the *X*-axis goes along the incident point to the exit point, and the *Y*-axis direction is determined according to the right-hand rule.

Through the CAM simulation software, we can easily get the position and normal information of the discrete points on the surface of the workpiece in the workpiece coordinate system. Considering the location parameters of transducer installation, scanning path and the requirements of incident or exit direction of ultrasonic wave, the purpose of coordinate transformation is to transform the position and normal direction information of discrete points and the deflection angle of transducer into the position and posture of trajectory points in the coordinate system of the robots.

For a space vector *P*, let its position in the coordinate system {A}, {B}, {C}, and {W} be expressed as ^A^ *P*, ^B^ *P*, ^C^ *P*, and ^W^ *P*, respectively. According to the principle of robot kinematics, we need to identify the posture of transducers in the coordinate system of the robot {W} based on the pose information in the Cartesian coordinate system of workpiece {A}. According to the principle of coordinate transformation,

(6) PW=TAWTCAPC,

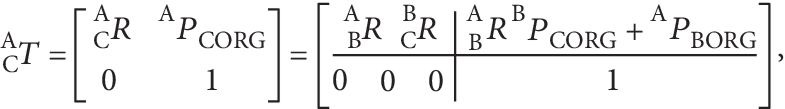
(7)where

(8) RBA=ξXξYξZφXφYφZψXψYψZ,

[*ξ*_*X*_, *ξ*_*Y*_, *ξ*_*Z*_] is the direction vector of the *X*-axis of coordinate system {B} in the coordinate system {A}; *φ* = [*φ*_*X*_, *φ*_*Y*_, *φ*_*Z*_] is the direction vector of the *Y*-axis of coordinate system {B} in the coordinate system {A}; and *ψ* = [*ψ*_*X*_, *ψ*_*Y*_, *ψ*_*Z*_] is the direction vector of the *Z*-axis of coordinate system {B} in the coordinate system {A}. Supposing that the location of the incident point is *P*_i_ and the location of the out point is *P*_o_ at some time, their positions and normal directions in the coordinate system {A} can be expressed as

(9) PiA=xi,yi,zi,nxi,nyi,nziT,PoA=xo,yo,zo,nxo,nyo,nzoT,

where [*x*, *y*, *z*] represents the position information and [*nx*, *ny*, *nz*] represents the normal vector.

According to the definition of axes in coordinate system {B},

(10) $$\begin{array}{c} \psi =\left[\psi_{X},\psi_{Y},\psi_{Z}\right]=\left[{nx}_{\text{i}},{ny}_{\text{i}},{nz}_{\text{i}}\right],\\ \varphi =\left[\varphi_{X},\varphi_{Y},\varphi_{Z}\right]=\frac{\psi \times \left[x_{\text{o}}-x_{\text{i}},y_{\text{o}}-y_{\text{i}},z_{\text{o}}-z_{\text{i}}\right]}{\left|\psi \times \left[x_{\text{o}}-x_{\text{i}},y_{\text{o}}-y_{\text{i}},z_{\text{o}}-z_{\text{i}}\right]\right|},\\ \xi =\varphi \times \psi =\frac{\psi \times \left[x_{\text{o}}-x_{\text{i}},y_{\text{o}}-y_{\text{i}},z_{\text{o}}-z_{\text{i}}\right]\times \left[{nx}_{\text{i}},{ny}_{\text{i}},{nz}_{\text{i}}\right]}{\left|\psi \times \left[x_{\text{o}}-x_{\text{i}},y_{\text{o}}-y_{\text{i}},z_{\text{o}}-z_{\text{i}}\right]\right|}. \end{array}$$

In order to ensure that the focal point of the receiving transducer coincides with the measured point and that the acoustic axis deviates from the *θ*_cr_ of the incident point normal within the plane formed by the incident axis and the propagation path, the measured discrete coordinate system {B} and transducer coordinate system {C} should satisfy the following relationship:

(11) RCB=1000−1000−1cosθcr0−sinθcr010sinθcr0cosθcr=cosθcr0sinθcr0−10sinθcr0−cosθcr,

(12) PCORGB=0.

Bring equations (8), (11), and (12) into equation (7):

(13) RCA=ξXξYξZφXφYφZψXψYψZcosθcr0sinθcr0−10sinθcr0−cosθcr=ξXcθcr+ξZsθcr−ξYξXsθcr−ξZcθcrφXcθcr+φZsθcr−φYφXsθcr−φZcθcrψYcθcr+ψYsθcr−ψYψYsθcr−ψYcθcr,PCORGA=PCORGA=xiyizi.

**cos** and **sin** are abbreviated as **c** and **s**, respectively.

(14) RCW=RAWRCA=RAWξXcθcr+ξZsθcr−ξYξXsθcr−ξZcθcrφXcθcr+φZsθcr−φYφXsθcr−φZcθcrψYcθcr+ψYsθcr−ψYψYsθcr−ψYcθcr,

(15) PCORGW=RAWPCORGA+PAORGW=RAWxiyizi+PAORGW.

Although the workpiece coordinate system {A} is unknown, its position and posture is fixed in the detection process. Therefore, we can calibrate the coordinate system {A} by several characteristic points to determine the transformation matrix _A_^W^ *R* and ^W^ *T*_AORG_ between the workpiece coordinate system {A} and the reference coordinate system of the robot {W}. Bringing the _A_^W^ *R* and ^W^ *T*_AORG_ into equations 14 and 15, we can calculate the position and posture of the excitation transducer in coordinate {W}.

The pose determination method of the receiving transducer is similar to that of the transmitting transducer. The only differences between them are the establishment of coordinate system {B} and the deflection direction of transducer.

In this case,

(16) ψ=ψX,ψY,ψZ=nxo,nyo,nzo,φ=φX,φY,φZ=ψ×xi−xo,yi−yo,zi−zoψ×xi−xo,yi−yo,zi−zo,ξ=φ×ψ=ψ×xi−xo,yi−yo,zi−zonxo,nyo,nzoψ×xi−xo,yi−yo,zi−zo,RCA=ξXξYξZφXφYφZψXψYψZcos−θcr0sin−θcr0−10sin−θcr0−cos−θcr,PCORGA=PBORGA=xoyozo.

## 3. Results and Discussion

**Figure 7:**
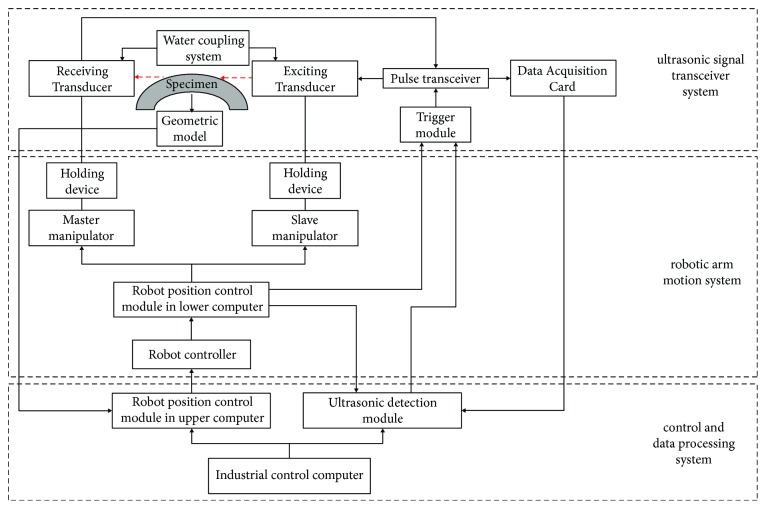
Ultrasonic measuring robot system.

Ultrasonic measuring robot for residual stress of complex surface components includes hardware system and software system. As shown in Figure 7, the hardware system is mainly composed of three parts: the robotic arm motion mechanism, the ultrasonic signal transceiver system, and the control and data processing system. Two six-DOF industrial robots are used to implement the gripping, position, and posture control of the ultrasonic transducers and automatic scanning; the ultrasonic signal transceiver system mainly includes a pulse transceiver, a high-frequency data acquisition card, two ultrasonic transducers, and a water coupling system. The role of this system is to excite and receive ultrasonic signals at the detection location. The control and processing system is the “brain” of the entire measuring robot, realizing the core tasks of motion control and ultrasonic signal processing.

**Figure 8:**
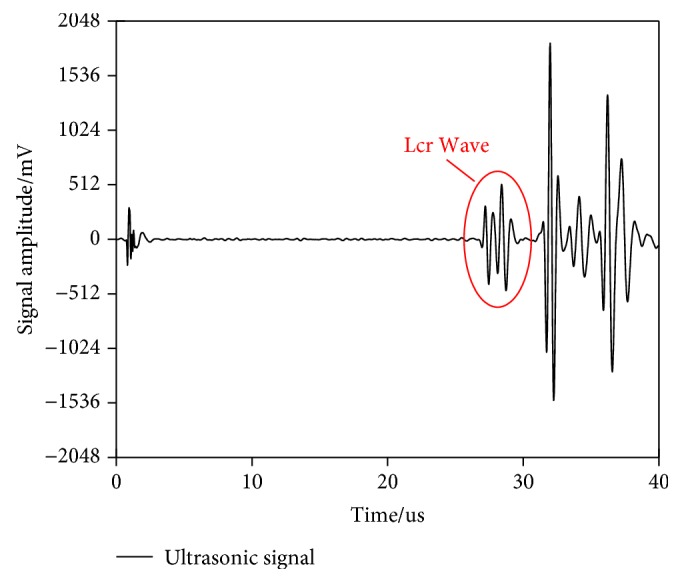
Ultrasonic signal.

The software system consists of the upper computer software subsystem and the lower computer software subsystem. The two subsystems cooperate with each other to jointly perform functions such as communication and control of the ultrasonic signal transceiver system and the manipulator movement system. The upper computer software is implemented in the industrial control computer to complete the trajectory planning and coordinate transforming. Another core task of the upper computer software is to process the ultrasonic transducer posture data and ultrasonic signals to obtain residual stress results. The ultrasonic signal is shown in Figure 8. The lower computer software refers to the software system implemented in the controller of the robot and is mainly responsible for robot motion control, external triggering ultrasonic pulse transceiver, and reading and transferring the robot position information.

**Figure 9:**
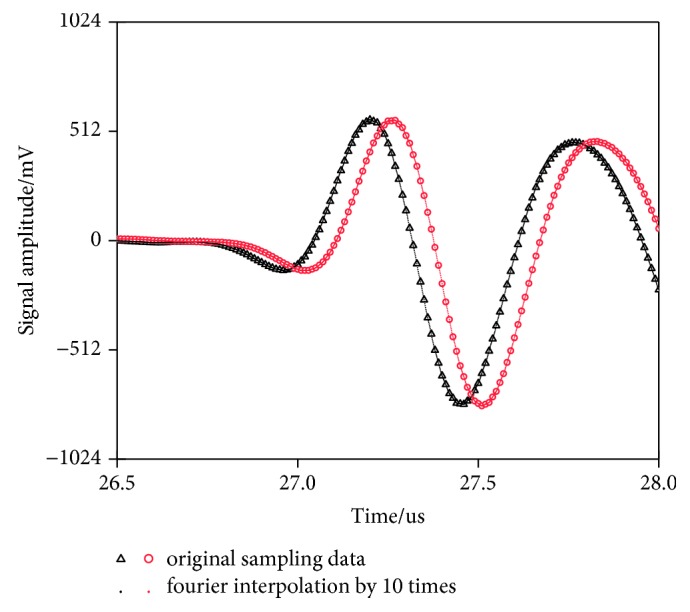
Ultrasonic signals before and after interpolation in different stress states.

**Figure 10:**
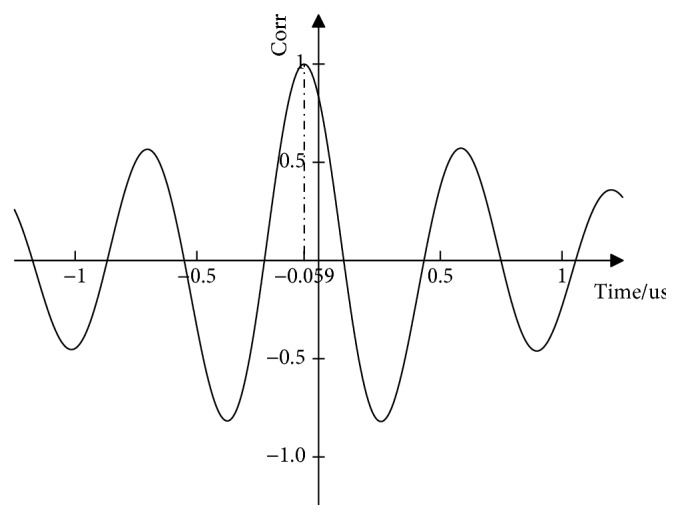
Cross-correlation of ultrasonic signals under two different stress states.

According to equation 5, the time difference directly determines the accuracy of the results. For acoustic time method of measuring residual stress, we are only interested in the effect of stress on the ultrasonic signal in the time domain. Hence, as shown in Figures 9 and 10, we combine interpolation and time-delayed autocorrelation theory to improve the accuracy of time measurement and calculate the delay of two sets of signals.

## 4. Conclusions

In this paper, a new ultrasonic measuring robot system with two manipulators was designed, which can realize the nondestructive automatic detection of the residual stress in the surface of a complex curved surface component by using critical refracted longitudinal wave.

1. Deduced an ultrasonic measuring principle and calculation formula of residual stress in curved surface components
2. Proposed a trajectory planning strategy for the robot with two manipulators
3. Established a coordinate transformation formula between the workpiece coordinate system and the robot coordinate system in consideration of the first critical angle
4. A system architecture of ultrasonic measuring robot for residual stress in complex curved surface components was proposed

## Data Availability

The data used to support the findings of this study are available from the corresponding author upon request.
